# Epidemiological and Clinical Characteristics of Interstitial Lung Diseases in Kuwait: A 9-Year Single-Center Observational Prospective Study

**DOI:** 10.1159/mpp/adpag005

**Published:** 2026-09-21

**Authors:** Mousa Elias Khadadah, Abdulaziz Taleb Muqim, Ali H. Ziyab, Mahmoud M. Elhosiny

**Affiliations:** a Department of Medicine, College of Medicine, Kuwait University, Jabriya, Kuwait; b Respiratory Division, Internal Medicine Department, Mubarak Al Kabeer Hospital, Ministry of Health, Jabriya, Kuwait; c Department of Community Medicine and Behavioral Sciences, College of Medicine, Kuwait University, Jabriya, Kuwait; d Department of Chest Medicine, Faculty of Medicine, Mansoura University, Mansoura, Egypt

**Keywords:** Hypersensitivity pneumonitis, Idiopathic pulmonary fibrosis, Interstitial lung disease, Lung fibrosis

## Abstract

**Objective:**

The data on epidemiological features of interstitial lung diseases (ILD) in the Gulf region are scarce. The aim of this study was to describe the baseline clinical and epidemiological characteristics of ILD patients in a single specialized expertise center in Kuwait.

**Subjects and Methods:**

This prospective, observational registry evaluated 167 patients newly diagnosed with ILD via a multidisciplinary team approach between November 2016 and October 2025. The demographics, exposures, comorbidities, and physiological data were systematically collected. Appropriate parametric and nonparametric descriptive statistics were applied to summarize and compare clinical variables across ILD subtypes.

**Results:**

Majority of the patients were male (60.5%), with a mean age of 58.7 years (SD ± 13.2). Idiopathic pulmonary fibrosis (IPF) was the most frequent subtype (34.1%), followed by connective tissue disease-related ILD (CTD-ILD; 23.4%) and hypersensitivity pneumonitis (HP; 20.4%). The environmental and occupational exposures were documented in 41.3% and 10.2% of the cohort, respectively, with environmental exposure predominant in the HP group (76.5%). Overall, gastroesophageal reflux disease was the most prevalent comorbidity (in 58.1% of cases). Patients with HP demonstrated the poorest baseline lung function compared to other subtypes, with a mean predicted forced vital capacity of 59.2% (SD ± 14.4).

**Conclusion:**

This 9-year prospective study provides the first real-world ILD dataset from Kuwait. IPF, CTD-ILD, and HP are the predominant clinical phenotypes. Knowledge of comprehensive exposure histories is vital for diagnostic evaluation to identify manageable HP before irreversible fibrosis develops.

**Highlights of the Study**

- Idiopathic pulmonary fibrosis is the most frequent interstitial lung disease subtype identified in this Kuwaiti cohort.
- Significant environmental exposure is present in more than 75% of patients with confirmed hypersensitivity pneumonitis.
- Gastroesophageal reflux disease is the most prevalent baseline clinical comorbidity observed across all interstitial lung disease subtypes.

## Introduction

Interstitial lung diseases (ILDs), also referred to as diffuse parenchymal lung diseases, comprise a heterogeneous group of over 200 chronic disorders characterized by varying degrees of inflammation and fibrosis within the lung parenchyma [1–3]. Despite their diverse etiologies, these conditions are grouped together due to overlapping clinical features, pathophysiological mechanisms, and radiological patterns [1, 2]. Over the past 15 years, the clinical approach to ILDs has entered a new era. International classification guidelines have evolved substantially, most notably by establishing a clear distinction between fibrotic and non-fibrotic ILD phenotypes to better guide clinical management [4].

Concurrently, the diagnostic paradigm has shifted away from invasive histopathological interventions toward a noninvasive, multidisciplinary consensus framework involving ILD-specialized pulmonologists, chest radiologists, thoracic pathologists, and rheumatologists [1, 5, 6]. This multidisciplinary team (MDT) approach is now considered the gold standard for navigating the complex diagnostic landscape of ILDs. Alongside these diagnostic advances, the therapeutic landscape has been transformed by the evolution of targeted anti-fibrotic medications. These therapies have fundamentally altered treatment protocols by significantly slowing the decline of forced vital capacity (FVC) in progressive fibrotic ILDs, including idiopathic pulmonary fibrosis (IPF) [7, 8].

The underlying etiology of ILD remains highly variable. Approximately two-thirds of cases are idiopathic, with IPF and idiopathic nonspecific interstitial pneumonia (iNSIP) being the most prevalent subtypes [6, 9]. Other forms arise from occupational or environmental exposures, such as hypersensitivity pneumonitis (HP), or secondary to connective tissue diseases (CTDs). Given this heterogeneity, utilizing prospective disease registries is crucial to characterizing real-world disease behavior, evaluating long-term treatment outcomes, and understanding regional variations in clinical phenotypes.

To date, epidemiological data regarding ILD in the Arabian Gulf region remain critically scarce. To address this knowledge gap, the Respiratory Division within the Department of Internal Medicine at Mubarak Al Kabeer Hospital (Ministry of Health, Kuwait) launched the Kuwait ILD Registry in November 2016. This single-center, prospective, observational, non-interventional registry represents the first dedicated ILD dataset from Kuwait. By analyzing the epidemiological trends and clinical characteristics of this cohort, this registry aims to optimize local standards of care, inform regional clinical protocols, and provide a foundational framework to facilitate future collaborative research across the Gulf region.

## Subjects and Methods

### Study Design and Setting

The Kuwait registry of ILD is a single-center, prospective, non-interventional cohort study that included all patients who were initially suspected or diagnosed with ILDs other than pulmonary sarcoidosis. The registry includes patients managed at the Respiratory Division of the Department of Internal Medicine at Mubarak Al Kabeer Hospital (Ministry of Health, Kuwait) – a principal tertiary expertise center. Recruitment spanned nearly 9 years, between November 29, 2016 and October 15, 2025.

The study was conducted in strict accordance with the ethical guidelines of the Declaration of Helsinki.

### Selection of Patients

Patients were recruited from healthcare polyclinics, respiratory, medical, and rheumatology outpatient clinics, and inpatient wards. Patients from other general hospitals and from private healthcare facilities in Kuwait were also accepted. Eligible patients were prospectively followed from the time of their initial visit until their last visit to the clinic, death, lung transplantation, or loss of follow-up. Information about the cause of death was obtained from their medical records.

The following criteria were utilized to define progression of ILD over 1 year: absolute changes in the forced vital capacity (FVC%) predicted into improvement (FVC increase ≥5%), stable (FVC decline or increase <5%), and significant or moderate progression (FVC decline >10% or FVC decline 5%–10%), respectively [10]. The initial visit was selected as the zero reference time. Since 2022, we have been using updated American Thoracic Society and the European Respiratory Society (ATS/ERS) criteria to define progressive pulmonary fibrosis [11].

Patients were eligible for enrollment if they met the following inclusion criteria: (1) aged above 18 years at the time of enrollment; (2) presented with a new-onset suspicion or confirmed diagnosis of ILD based on high-resolution computed tomography (HRCT) features; and (3) provided written informed consent for longitudinal registry follow-up.

Exclusion criteria consisted of: (1) secondary parenchymal changes due to a history of previous tuberculosis with post-tuberculous fibrotic sequelae, or current active pulmonary tuberculosis; (2) stable, steroid-responsive pulmonary sarcoidosis (advanced sarcoidosis patients presenting with irreversible, progressive lung fibrosis were eligible); and (3) confirmed COVID-19-associated interstitial changes with post-viral radiological evidence of fibrosis.

### Data Collection and Diagnostic Protocol

At the baseline enrollment visit, clinicians collected an exhaustive clinical history encompassing detailed demographics, occupational and environmental exposures (including specific avian, mold, and chemical vectors), systemic symptoms suggestive of CTDs, and family history. A comprehensive clinical examination followed by a complete functional assessment was conducted using complete pulmonary function testing, which measured FVC, forced expiratory volume in the first second (FEV_1_), total lung capacity (TLC), and diffusing capacity of the lung for carbon monoxide (DLCO) via the MasterScreen-PFT system (CareFusion, Hoechberg, Germany). Values were calibrated daily according to manufacturer protocols and interpreted using the ATS/ERS guidelines [12]. Functional exercise capacity was assessed via the 6-min walk distance test. Comprehensive laboratory evaluations included complete blood counts, hepatic and renal metabolic panels, and an extensive autoimmune serological workup. HRCT examinations were performed for all patients utilizing a uniform 64-slice Multidetector Computed Tomography scanner (GE Healthcare, USA). Diagnostic bronchoscopy with bronchoalveolar lavage using a video bronchoscope (Olympus Medical Systems, Tokyo, Japan) was performed whenever clinically indicated.

A final diagnosis for each patient was established through a rigorous MDT framework. The core MDT comprised three expert pulmonologists, a specialized thoracic radiologist, and an assigned clinical rheumatologist. A thoracic pathologist was integrated into the discussion when histopathological specimens were available. Diagnostic categorization followed the evolving consensus statements of the ATS/ERS Multidisciplinary International Consensus Classification of Idiopathic Interstitial Pneumonias [11, 13, 14]. For cases presenting with typical clinical features and highly characteristic HRCT findings, the MDT achieved a confident diagnosis without requiring a surgical lung biopsy. Conversely, in alignment with clinical practice guidelines, MDT evaluation was considered essential when the HRCT pattern was probable or indeterminate for usual interstitial pneumonia (UIP), when alternative etiologies were suspected, or when clinical, radiological, and pathological data were discordant. Connective tissue disease was established according to the American College of Rheumatology (ACR) criteria. Interstitial pneumonia with autoimmune features (IPAF) was diagnosed when features of autoimmunity were present but did not satisfy the full American College of Rheumatology and the European Alliance of Associations for Rheumatology (ACR/EULAR) criteria for a specific connective tissue disorder.

Given the long inclusion window (2016–2025), patients’ diagnostic classifications were dynamically reviewed and cross-referenced with updated clinical practice guidelines – specifically the 2018 and 2022 ATS/ERS guidelines [11, 14], to ensure continuous phenotypic uniformity across the study duration. While longitudinal progression data were recorded, the primary endpoint of the present analysis remains constrained to characterizing baseline clinical, demographic, and physiological metrics.

### Statistical Analysis

Statistical analyses were performed using SAS 9.4 (SAS Institute, Cary, NC, USA). The statistical significance level was set at α = 0.05 for all analyses. The normality of continuous variables was evaluated using the Shapiro-Wilk test plus visual inspection of histograms and Q-Q plots. Descriptive statistics included frequencies and proportions for categorical variables, while continuous variables were summarized using means (standard deviation) for normally distributed data or medians (interquartile range) for non-normally distributed data. Univariable associations between ILD subtypes and categorical variables were evaluated using the chi-squared (χ^2^) test. Differences in continuous outcomes across ILD subtypes were assessed using one-way analysis of variance (ANOVA) for normally distributed variables and the Kruskal-Wallis test for non-normally distributed variables.

## Results

This study reports baseline data from 167 ILD patients enrolled in the registry from November 29, 2016, to October 15, 2025, at a single center in Kuwait. The patients’ characteristics in the total sample and in the ILD subtype are presented in Table 1. In the total sample, there were more male patients (60.5%) than female patients (39.5%). The mean age of the analyzed patients was 58.7 (standard deviation: ± 13.2) years, with very few patients aged ≤34 years (3.6%) and 35–44 years (11.4%). Ever cigarette smoking was reported in 46.2% of the patients. Moreover, occupational and environmental exposures were reported in 10.2% and 41.3% of patients, respectively. Most referrals were made by pulmonologists (59.3%), followed by rheumatologists (19.1%; Table 1).

**Table 1.** Characteristics of patients with interstitial lung disease (ILD) in the total study sample (*n* = 167) and according to ILD subtype

​		Subtype of interstitial lung disease (ILD), *n* (%)				
variable	all (*n* = 167), *n* (%)	CTD–ILD (*n* = 39)	HP (*n* = 34)	IPF–UIP (*n* = 57)	NSIP (*n* = 19)	other (*n* = 18)
Sex	​	​	​	​	​	​
Male	101 (60.5)	12 (30.8)	22 (64.7)	47 (82.5)	8 (42.1)	12 (66.7)
Female	66 (39.5)	27 (69.2)	12 (35.3)	10 (17.5)	11 (57.9)	6 (33.3)
Age, years	​	​	​	​	​	​
Mean (SD)	58.7 (13.2)	52.8 (12.5)	59.3 (13.7)	64.2 (11.4)	61.8 (9.6)	49.6 (13.1)
Median (Q1, Q3)	60.0 (48.0, 68.0)	51.0 (44.0, 62.0)	59.5 (48.0, 69.0)	65.0 (59.0, 73.0)	63.0 (54.0, 70.0)	51.0 (43.0, 58.0)
≤34	6 (3.6)	1 (2.6)	2 (5.8)	0 (0.0)	0 (0.0)	3 (16.7)
35–44	19 (11.4)	9 (23.1)	4 (11.8)	3 (5.3)	1 (5.3)	2 (11.1)
45–54	37 (22.2)	13 (33.3)	7 (20.6)	7 (12.3)	4 (21.0)	6 (33.3)
55–64	41 (24.5)	8 (20.5)	7 (20.6)	17 (29.8)	5 (26.3)	4 (22.2)
65–74	47 (28.1)	7 (17.9)	10 (29.4)	19 (33.3)	8 (42.1)	3 (16.7)
≥75	17 (10.2)	1 (2.6)	4 (11.8)	11 (19.3)	1 (5.3)	0 (0.0)
Nationality	​	​	​	​	​	​
Kuwaiti	98 (58.7)	23 (59.0)	20 (58.8)	35 (61.4)	11 (57.9)	9 (50.0)
Non-Kuwaiti	69 (41.3)	16 (41.0)	14 (41.2)	22 (38.6)	8 (42.1)	9 (50.0)
Cigarette smoking status	​	​	​	​	​	​
Never	84 (53.8)	27 (75.0)	21 (61.8)	19 (36.5)	12 (66.7)	5 (31.3)
Ever	72 (46.2)	9 (25.0)	13 (38.2)	33 (63.5)	6 (33.3)	11 (68.7)
Missing, *n*	11	3	0	5	1	2
Occupational exposure	​	​	​	​	​	​
Yes	17 (10.2)	0 (0.0)	5 (17.2)	8 (15.1)	2 (10.5)	2 (12.5)
No	138 (82.6)	38 (100.0)	24 (82.8)	45 (84.9)	17 (89.5)	14 (87.5)
Missing, *n*	12	1	5	4	0	2
Environmental exposure	​	​	​	​	​	​
Yes	69 (41.3)	6 (15.4)	26 (76.5)	21 (36.8)	9 (47.4)	7 (38.9)
No	98 (58.7)	33 (84.6)	8 (23.5)	36 (63.2)	10 (52.6)	11 (61.1)
Duration of symptoms	​	​	​	​	​	​
<6 months	46 (27.5)	13 (33.3)	3 (8.8)	19 (33.3)	3 (15.8)	8 (44.4)
6–24 months	38 (22.8)	8 (20.5)	10 (29.4)	13 (22.8)	4 (21.0)	3 (16.7)
>24 months	83 (49.7)	18 (46.2)	21 (61.8)	25 (43.9)	12 (63.2)	7 (38.9)
Referral	​	​	​	​	​	​
Pulmonologist	99 (59.3)	6 (15.3)	32 (94.1)	37 (64.9)	11 (57.9)	13 (72.2)
Rheumatologist	32 (19.1)	31 (79.5)	0 (0.0)	0 (0.0)	1 (5.3)	0 (0.0)
General practitioner	13 (7.8)	1 (2.6)	0 (0.0)	8 (14.0)	3 (15.8)	1 (5.6)
Other	23 (13.8)	1 (2.6)	2 (5.9)	12 (21.1)	4 (21.0)	4 (22.2)

SD, standard deviation; Q1, first quartile; Q3, third quartile.

The most common subtype of ILD in our sample of patients was IPF-UIP (34.1%, *n* = 57), followed by CTD-ILD (23.4%, *n* = 39), HP (20.4%), NSIP (11.3%), and other subtypes of ILDs (10.8%; Fig. 1). The majority of patients with CTD-ILD (69.2%) and NSIP (57.9%) were females, while the majority of patients with HP (64.7%) and IPF-UIP (82.5%) were males (*p* < 0.001; Table 1). The mean age of patients differed by ILD subtype (*p* < 0.001), with IPF-UIP patients having the highest mean age (64.2 ± 11.4 years), while CTD-ILD patients had the lowest (52.8 ± 12.5). There was no statistically significant difference in the distribution of ILD subtypes by patient nationality (*p* = 0.946). Cigarette smoking was differentially distributed across ILD subtypes (*p* = 0.001), with the majority of IPF/UIP patients (63.5%) reporting a history of ever smoking. Environmental exposure was predominant among HP patients (76.5%), followed by NSIP patients (47.4%; *p* < 0.001; Table 1).

**Fig. 1.**
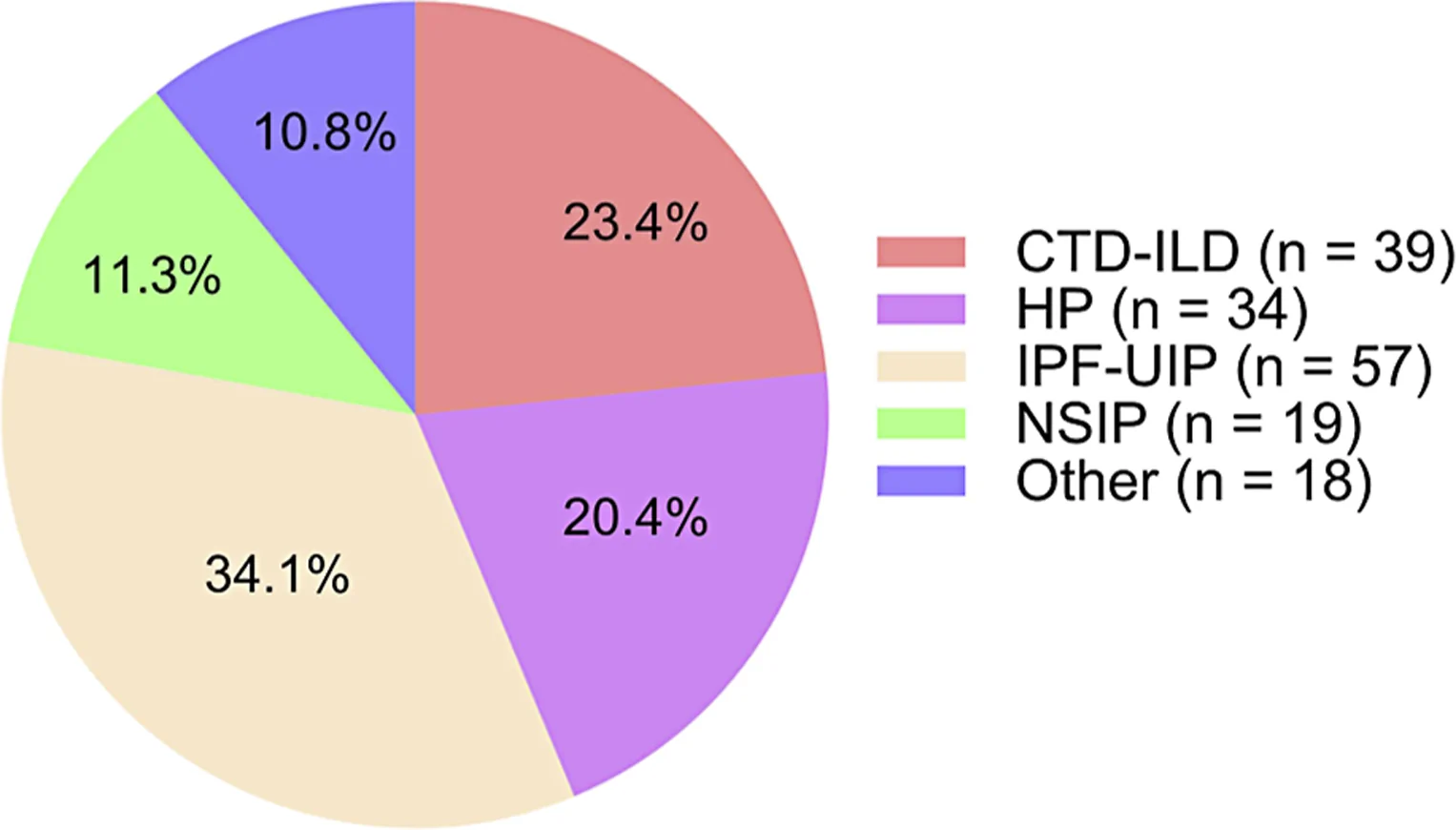
Distribution of interstitial lung disease (ILD) subtypes in the sample of 167 patients.

The data on comorbid conditions among the analyzed sample of ILD patients have been presented in Table 2. Gastroesophageal reflux disease (GERD) was the most reported comorbid condition (58.1%) among the total sample of patients. Dyslipidemia showed a differential distribution across ILD subtypes (*p* = 0.009), with NSIP patients reporting the highest prevalence (31.6%) compared with other ILD subtypes. Similarly, GERD was most common among NSIP patients (84.2%); nonetheless, there was no statistical evidence for a difference across the ILD subtypes in GERD prevalence (*p* = 0.129). Asthma was differentially distributed across ILD subtypes (*p* = 0.003), with HP patients reporting the highest preexisting asthma (38.2%) compared with the other ILD subtypes (Table 2). GERD, bronchial asthma, pulmonary hypertension, and diabetes mellitus ranked among the top four associated comorbidities (58.1%, 18.6%, 18.0%, and 17.4%, respectively); however, they did not show differential distribution across ILD subtypes (Table 2).

**Table 2.** Preexisting comorbid conditions in patients with interstitial lung disease (ILD) in the total study sample (*n* = 167) and according to ILD subtype

​	​	Subtype of interstitial lung disease (ILD), *n* (%)					​
condition	all (*n* = 167), *n* (%)	CTD–ILD (*n* = 39)	HP (*n* = 34)	IPF–UIP (*n* = 57)	NSIP (*n* = 19)	other (*n* = 18)	*p* value^a^
Diabetes mellitus	29 (17.4)	6 (15.4)	7 (20.6)	7 (12.3)	6 (31.6)	3 (16.7)	0.257
Dyslipidemia	15 (9.0)	1 (2.6)	3 (8.8)	4 (7.0)	6 (31.6)	1 (5.6)	0.009
GERD	97 (58.1)	24 (61.5)	18 (52.9)	33 (57.9)	16 (84.2)	6 (33.3)	0.129
Asthma	31 (18.6)	9 (23.1)	13 (38.2)	4 (7.0)	4 (21.1)	1 (5.6)	0.003
COPD	26 (15.6)	3 (7.7)	2 (5.9)	12 (21.1)	3 (15.8)	6 (33.3)	0.135
Pulmonary hypertension^b^	23 (18.0)	5 (16.7)	8 (29.6)	5 (11.4)	4 (26.7)	1 (8.3)	0.221

GERD, gastroesophageal reflux disease; COPD, chronic obstructive pulmonary disease.

^a^ Calculated using chi-squared test. If any cell expected count was less than 5 observations, Fisher’s exact test was used. The “other” disease subtype was excluded when evaluating associations between ILD subtypes and comorbid conditions.

^b^ The total number of participants with available information for pulmonary hypertension is 128.

The distribution of signs and symptoms observed in the studied patient sample is shown in Table 3. Dyspnea (94.0%) and cough (85.0%) were highly common among the ILD patients, followed by clubbing (31.1%). Clubbing was most common among IPF-UIP (40.4%) and HP (35.3%) of the patients. Raynaud’s hands were observed in 9 patients, all of whom were classified as having CTD-ILD.

**Table 3.** Signs and symptoms in patients with interstitial lung diseases (ILD) in the total study sample (*n* = 167) and according to ILD subtype

​	​	Subtype of interstitial lung disease (ILD), *n* (%)					​
signs/symptoms	all (*n* = 167), *n* (%)	CTD–ILD (*n* = 39)	HP (*n* = 34)	IPF–UIP (*n* = 57)	NSIP (*n* = 19)	other (*n* = 18)	*p* value^a^
Clubbing	52 (31.1)	10 (25.6)	12 (35.3)	23 (40.4)	2 (10.5)	5 (27.8)	0.074
Raynaud’s	9 (5.4)	9 (23.1)	0 (0.0)	0 (0.0)	0 (0.0)	0 (0.0)	<0.001
Dyspnea	157 (94.0)	38 (97.4)	34 (100.0)	50 (87.7)	18 (94.7)	17 (94.4)	0.079
Cough	142 (85.0)	30 (76.9)	30 (88.2)	50 (87.7)	17 (89.5)	15 (83.3)	0.469
Hemoptysis	2 (1.2)	0 (0.0)	1 (2.9)	0 (0.0)	0 (0.0)	1 (5.6)	0.356
Wheezing	13 (7.8)	2 (5.1)	3 (8.8)	4 (7.0)	1 (5.3)	3 (16.7)	0.965

^a^ Calculated using chi-squared test. If any cell expected count was less than 5 observations, Fisher’s exact test was used. The “other” disease subtype was excluded when evaluating associations between ILD subtypes and signs/symptoms.

Lung function parameters and walking test data are presented in Table 4. Overall, HP patients demonstrated the poorest lung function parameters compared with patients with other ILD subtypes. For instance, HP patients had lower predicted FVC on average than NSIP patients (age- and sex-adjusted means: 59.7% vs. 71.0%, *p* value = 0.032; Fig. 2). Similarly, predicted FEV_1_ was lower in HP patients than in NSIP patients (age- and sex-adjusted means: 62.1% vs. 75.9%, *p* value = 0.013; Fig. 2). Moreover, the predicted TLC was lower in HP patients (age- and sex-adjusted mean: 57.5%) than in IPF-UIP (age- and sex-adjusted mean: 71.3%; *p* value = 0.002) and NSIP (age- and sex-adjusted mean: 73.9%; *p* value = 0.003; Fig. 2).

**Table 4.** Lung function parameters (% predicted) and 6-min walk test in the total sample of patients with interstitial lung disease (ILD) and according to ILD subtypes

​			Subtype of interstitial lung disease (ILD), Mean (SD)										​
	all patients		CTD–ILD		HP		IPF–UIP		NSIP		other		
	*n*	mean (SD)	*n*	mean (SD)	*n*	mean (SD)	*n*	mean (SD)	*n*	mean (SD)	*n*	mean (SD)	*p* value
FVC	156	65.7 (17.8)	37	64.9 (19.2)	31	59.2 (14.4)	53	66.3 (17.8)	18	71.7 (18.3)	17	70.5 (18.2)	0.159^a^
FEV_1_	155	68.3 (18.9)	37	67.4 (19.5)	31	61.7 (15.8)	53	69.5 (19.3)	18	76.7 (19.8)	16	69.5 (17.9)	0.086^a^
TLC	143	67.1 (18.6)	36	65.1 (18.7)	28	56.8 (17.1)	47	70.4 (17.1)	17	74.3 (17.1)	15	72.8 (20.8)	0.005^a^
DLCO^b^	129	42.8 (21)	31	43.0 (34.0)	26	37.5 (23.5)	43	41.2 (19.0)	15	42.0 (23.2)	14	49.0 (21)	0.756^c^
6-min walk distance, m	122	355.8 (132.6)	33	340.5 (135.7)	24	369.8 (140.4)	40	363.7 (130.1)	12	324.1 (140.9)	13	374.1 (121.0)	0.580^a^
Lowest SpO2 during walk^b^	122	89.5 (12.0)	33	91.0 (13.0)	24	85.5 (7.0)	40	89.5 (11)	12	91.0 (6.5)	13	90.0 (16.0)	0.106^c^

FVC, forced vital capacity; FEV_1_, forced expiratory volume in the first second; TLC, total lung capacity; DLCO, diffusing capacity of the lungs for carbon monoxide; SpO2, peripheral oxygen saturation.

^a^ Calculated using analysis of variance (ANOVA).

^b^ Reporting median (interquartile range [IQR]: Q3–Q1) due to non-normal distribution.

^c^ Calculated using the Kruskal-Wallis test.

**Fig. 2.**
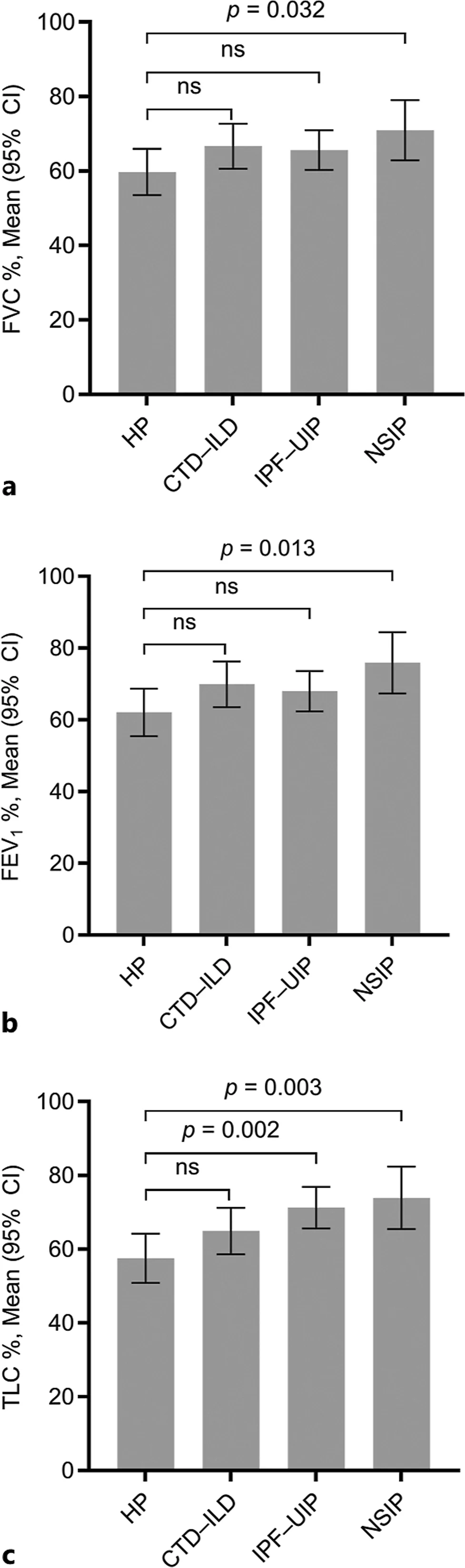
Age- and sex-adjusted lung function parameters according to interstitial lung disease (ILD) subtypes. **a** Levels of predicted forced vital capacity (FVC) according to ILD subtypes. **b** Levels of predicted forced expiratory volume in 1 s (FEV1) according to ILD subtypes. **c** Levels of predicted total lung capacity (TLC) according to ILD subtypes. ns, not statistically significant.

## Discussion

ILDs comprise a highly heterogeneous spectrum of more than 200 distinct pathological entities characterized by varying degrees of alveolar inflammation and parenchymal fibrosis. Despite substantial contemporary leaps in elucidating their respective underlying etiologies, establishing an accurate diagnosis remains an intricate clinical challenge [15]. It requires sophisticated coordination among expert pulmonologists, thoracic radiologists, rheumatologists, and histopathologists. Accumulating global evidence underscores that the definitive epidemiological patterns, phenotypic distributions, and diagnostic frequencies of ILDs are subject to profound geographical, environmental, and socio-demographic variations. Prospective disease registries serve as indispensable real-world clinical tools. They offer invaluable insights regarding localized disease behavior, natural history trajectories, phenotypic progression, and baseline characteristics, which collectively guide regional healthcare resource allocation and preventative clinical protocols [16]. The present study represents a prospective descriptive analysis tracking a well-characterized cohort over a 9-year period. To our knowledge, this work reports the first dedicated dataset detailing baseline ILD patterns within the State of Kuwait and stands as only the second comprehensive registry infrastructure within the Arabian Gulf region.

In our cohort, IPF-UIP emerged as the most prevalent ILD classification, accounting for 34.1% of all cases. This predominant distribution aligns closely with European and transcontinental registry data. For instance, the Research Burden of Interstitial Lung Diseases (RBILD) registry in Turkey reported IPF as its leading subclass at 30.5% [17], while alternative registries frequently position it as either the primary or secondary most prevalent fibrotic condition after accounting for sarcoidosis [18], the stable forms of which were deliberately excluded from our registry footprint to isolate progressive parenchymal processes. Similarly, data from the Canadian Registry for Pulmonary Fibrosis (CARE-PF) ranked IPF as a dominant cohort feature (24.7%) [19].

This high diagnostic yield of IPF within our center can be systematically attributed to several factors. First, the progressive refinement and widespread clinical implementation of the international ATS/ERS guidelines have significantly harmonized diagnostic thresholds. HRCT features of a definitive UIP pattern are now recognized as highly specific, enabling a confident noninvasive diagnosis in a clinically compatible host without the mandatory requirement of a surgical lung biopsy. Second, our cohort was characterized by an older demographic (mean age: 58.7 ± 13.2 years) and a notable male predominance (60.5%), paired with a high prevalence of well-established fibrotic risk factors such as cigarette smoking (46.2%) and GERD (58.1%).

Conversely, our findings diverge markedly from the prospective Indian ILD registry, where IPF constituted only 13.7% of the study population [20]. This variation is likely driven by historical differences in data collection timelines, environmental exposure variations, and the application of older diagnostic paradigms, such as the 2011 ATS/ERS guidelines, which mandated stricter criteria for non-biopsied patients [21]. More broadly, recent global epidemiological reviews highlight that while Western countries mirror our higher proportions of IPF, developing regions often showcase an inflation of exposure-driven or granulomatous ILDs, highlighting the fluid nature of global ILD epidemiology [22–24].

Connective tissue disease-associated ILD (CTD-ILD) represented the second most frequent diagnostic subclass in our registry, encompassing 23.4% of the cohort. The comparative literature shows substantial variance regarding this phenotype. In a single-center registry from Saudi Arabia, CTD-ILD emerged as the single most common classification. However, that cohort utilized a broader definition that included “lung-dominant CTD” for patients who did not fully satisfy the formal ACR classification criteria [25], which potentially inflated their final numbers [16]. Our strict adherence to the consensus ACR/EULAR criteria and the targeted application of the IPAF framework ensured a highly specific diagnostic yield. Our data also demonstrated a classic demographic profile for this subclass, with females comprising 69.2% of the CTD-ILD group, reinforcing the established epidemiological reality that auto-immune-mediated parenchymal injury predominantly impacts women [26].

HP constituted the third most frequent diagnosis within our cohort at 20.4%, showing close parity with Turkish (third most frequent) [17] and Saudi datasets [16]. This stands in sharp, illustrative contrast to the Indian registry, where HP overwhelmingly dominated at 47.3% [20], and an Egyptian single-center study from the Minia Governorate, which recorded an HP prevalence of 51% [27]. To understand these geographical differences, one must analyze localized cultural and environmental factors. Minia Governorate is a highly rural, agricultural region bordering a network of agrarian villages where domestic avian husbandry and livestock handling are heavily integrated into daily domestic life, exposing up to 98% of their cohort to organic antigens. In India, the high HP burden was primarily attributed to mold and fungal proliferation within domestic evaporative air-cooler pads.

In our cohort, the driving antigen vector is fundamentally distinct. Bird breeding, specifically the keeping of pigeons, canaries, and parrots, is a highly popular, culturally rooted hobby in Kuwait. Previous cross-sectional data indicate that nearly 30% of households in Kuwait keep domestic birds, highlighting a highly prevalent local source of avian antigen exposure [28]. This practice spans domestic properties, suburban chalets, and frequent weekend visits to agricultural livestock farms. To evaluate this further, we performed a quantitative sub-analysis of our exposure data. We confirmed that out of the 69 patients presenting with positive environmental histories, 61 (88.4%) reported direct, sustained exposure to avian antigens. Interestingly, when stratified by nationality, there was no statistically significant difference in exposure rates: environmental exposure was documented in 41.8% of Kuwaiti citizens compared to 40.6% of non-Kuwaiti residents (*p* = 0.871; data not shown). This indicates that the environmental risk of avian exposure is uniformly distributed across the socioeconomic and cultural fabric of the country, irrespective of citizenship status.

The degree of pathogenic association between identifiable antigens and subsequent parenchymal disease behavior is highly variable. While a direct causal link is easily established in classic HP, the role of environmental exposures in apparently “idiopathic” diseases like IPF remains a subject of intense investigation. In our study, environmental exposures were reported in 36.8% of patients diagnosed with IPF, a finding that echoes data from the Canadian CARE-PF registry, which identified hidden environmental or domestic exposures in over 50% of its IPF cohort [19]. Our findings should be contextualized within the observations by Morell et al. [29], which demonstrated that a substantial proportion of patients initially fulfilling the diagnostic criteria for IPF are found to have occult, unrecognized domestic or avian antigen exposures upon meticulous exploration. When these exposures are removed, or patients are treated as chronic HP, their clinical courses deviate significantly from typical idiopathic trajectories. Hence, the observed baseline association in our cohort underscores the clinical need to conduct meticulous, exhaustive environmental and occupational histories in all ILD patients, as subtle exposures may mimic idiopathic processes or act as inciting agents that accelerate fibrotic pathways.

Among the co-morbidities captured within our 9-year registry, GERD, bronchial asthma, pulmonary hypertension, and diabetes mellitus ranked among the top four associated conditions. GERD was remarkably prevalent, affecting 58.1% of the total study population. This is consistent with international data, including the Canadian registry, which identifies gastric reflux as a prominent clinical co-factor [19]. Interestingly, GERD was exceptionally prevalent within our NSIP group (84.2%) and was highly represented in the HP group (52.9%). While the relationship between GERD and IPF-UIP via chronic microaspiration is well-documented, its high prevalence in HP has historically been less clear.

However, recent literature has shed light on this relationship; a study by Elkhatib et al. [30] demonstrates a bidirectional pathophysiological link between GERD and HP. Chronic microaspiration of gastric contents can induce severe epithelial injury and disrupt the alveolar-capillary barrier, thereby potentially lowering the threshold for sensitization to inhaled organic antigens and accelerating progressive fibrosing pathways. Given the cross-sectional, descriptive nature of our baseline dataset, these observations should be interpreted as associations rather than confirmed independent causal risk factors.

Additionally, we noted a high prevalence of preexisting bronchial asthma within the HP cohort (38.2%). This clinical intersection suggests that repeated exposure to organic avian antigens may initially manifest as reactive airway disease or asthma-like symptoms, potentially delaying the recognition of underlying parenchymal hypersensitivity until advanced, fixed structural deficits take hold.

The baseline physiological profiles captured in this registry revealed that patients with HP presented with the most severe functional impairment. The HP subgroup demonstrated significantly lower age- and sex-adjusted mean predicted FVC (59.2%), TLC (56.8%), and DLCO (37.5%) compared to other ILD cohorts. This severe restrictive defect is closely linked to our data on symptom duration: over 61.8% of our HP patients experienced progressive respiratory symptoms for more than 24 months before receiving an accurate diagnosis and starting targeted therapy. This prolonged diagnostic latency suggests that many HP patients are initially misdiagnosed or managed nonspecifically for obstructive airway diseases like asthma. Consequently, they miss the therapeutic window for early antigen avoidance and immunomodulatory intervention, presenting only after developing advanced, irreversible progressive pulmonary fibrosis.

Optimizing the diagnostic timeline for these complex conditions requires a well-MDT framework. Within this infrastructure, the role of the clinical rheumatologist is paramount. Rather than functioning as a downstream external consultant, the rheumatologist serves as an active, frontline investigator within our MDT. Their specialized expertise is essential for identifying subtle, subclinical systemic autoimmune features that may easily be overlooked during a standard respiratory exam. Furthermore, the rheumatologist is central to interpreting complex autoantibody panels and classifying borderline phenotypes like IPAF. This ensures that patients receive appropriately tailored immunomodulatory or anti-fibrotic regimens early in their disease course.

While this study offers comprehensive baseline insights into the ILD landscape in Kuwait, certain methodological limitations must be acknowledged. First, this registry is a single-center initiative based at a major regional government hospital. While this specialized center captures a substantial portion of complex respiratory referrals, the findings may not fully represent the entire ILD population across all governorates of Kuwait. However, given that Kuwait is a geographically small country with centralized, highly interconnected healthcare infrastructure, single-center data from a principal tertiary hub carries significant epidemiological weight. Second, the absolute sample size (*n* = 167) is modest when compared to large-scale international registries. Consequently, smaller subgroups are highly exploratory, and subsequent univariable statistical comparisons are likely underpowered, carrying an elevated risk of type II error.

Third, due to the real-world, non-interventional nature of the registry, screening echocardiography was not systematically performed across all participating patients, which could result in an underestimation of the true prevalence of secondary pulmonary hypertension in this population. Finally, recruitment across a long inclusion window (2016–2025) introduces temporal heterogeneity due to shifting international guidelines and diagnostic thresholds. While we attempted to control for this via ongoing MDT retrospective guideline cross-referencing, these shifts may have to some extent influenced historical patient categorization. In the absence of adjusted multivariable analysis, we explicitly caution against inferring direct causality regarding environmental or comorbid associations. These baseline data serve as an epidemiological foundation, and longitudinal analysis and outcome trajectories remain the focus of future analyses of this cohort.

## Conclusion

This 9-year prospective registry provides an important, real-world epidemiological dataset for ILDs in Kuwait, addressing a critical knowledge gap in the Arabian Gulf region. Our findings demonstrate that IPF-UIP, CTD-ILD, and HP are the dominant clinical phenotypes within this territory. It is essential to recognize that the percentages presented from this prospective cohort stem from a real-world, observational study design. Consequently, while these numbers are not rigid or “carved in stone,” they offer an accurate, clinically validated reflection of localized disease prevalence and comorbidity burdens. Ultimately, the high prevalence of advanced structural impairment among HP patients underscores the need for early diagnostic vigilance, where meticulous exposure history, particularly regarding regional avian antigens, remains a cornerstone to identifying manageable disease before irreversible fibrosis develops, a process further optimized by integrating a dedicated rheumatologist into the MDT framework.

## Acknowledgments

The support of the Department of Diagnostic Radiology, Mubarak Al Kabeer Hospital, and the Division of Rheumatology, Internal Medicine Department, Mubarak Al Kabeer Hospital is thankfully acknowledged. During the preparation of this manuscript, the authors used an artificial intelligence (AI)-assisted language model only for language editing and readability improvement. AI was used to enhance grammar, spelling, sentence structure, and overall clarity of the text. No scientific content, data, analyses, results, interpretations, references, or conclusions were generated by the AI tool. All intellectual content, including study conception, methodology, data collection, statistical analysis, and interpretation of findings, was performed by the authors. The authors reviewed and approved all edited text and assume full responsibility for the final content of the manuscript.

## Statement of Ethics

The study was approved by the local IRB (Institutional Research Board) of the Ministry of Health, Kuwait (code number: 2016/485). Informed consent was obtained from all participating patients or their representatives.

## Conflict of Interest Statement

The authors have no conflicts of interest to declare.

## Funding Sources

This study was not supported by any sponsor or funder.

## Author Contributions

Mousa Elias Khadadah, Abdulaziz Taleb Muqim, and Mahmoud M. Elhosiny contributed to the treatment of the patients. Mousa Elias Khadadah prepared the scientific rationale for the study. Abdullah Abdulredha AL-Sarraf and Abdelmohsen Eissa Ben Nakhi performed and interpreted the CT. Mousa Elias Khadadah, Abdulaziz Taleb Muqim, Ali H. Ziyab, and Mahmoud M. Elhosiny wrote the first draft of the manuscript. Mousa Elias Khadadah, Abdulaziz Taleb Muqim, Ali H. Ziyab, and Mahmoud M. Elhosiny revised the article critically for important intellectual content and approved the version to be published. Ali Hussain Ziyab performed the statistical analysis of the study. All authors read and approved the manuscript.

## Data Availability

The data that support the findings of this study are available on request from Mousa Elias Khadadah or the corresponding author.
